# Rational Design of Daunorubicin C-14 Hydroxylase Based on the Understanding of Its Substrate-Binding Mechanism

**DOI:** 10.3390/ijms24098337

**Published:** 2023-05-06

**Authors:** Jing Zhang, Ling-Xiao Gao, Wei Chen, Jian-Jiang Zhong, Chao Qian, Wen-Wen Zhou

**Affiliations:** 1 College of Biosystems Engineering and Food Science, Ningbo Research Institute, Zhejiang University, Hangzhou 310058, China; 2 School of Chemical and Biomolecular Engineering, The University of Sydney, Sydney, NSW 2006, Australia; 3 State Key Laboratory of Microbial Metabolism, School of Life Sciences and Biotechnology, Shanghai Jiao Tong University, Shanghai 200240, China; 4 College of Chemical and Biological Engineering, Zhejiang Provincial Key Laboratory of Advanced Chemical Engineering Manufacture Technology, Zhejiang University, Hangzhou 310027, China

**Keywords:** doxorubicin, daunorubicin, cytochrome daunorubicin C-14 hydroxylase, rational design strategy, molecular dynamics simulation

## Abstract

Doxorubicin is one of the most widely used antitumor drugs and is currently produced via the chemical conversion method, which suffers from high production costs, complex product separation processes, and serious environmental pollution. Biocatalysis is considered a more efficient and environment-friendly method for drug production. The cytochrome daunorubicin C-14 hydroxylase (DoxA) is the essential enzyme catalyzing the conversion of daunorubicin to doxorubicin. Herein, the DoxA from *Streptomyces peucetius* subsp. *caesius* ATCC 27952 was expressed in *Escherichia coli*, and the rational design strategy was further applied to improve the enzyme activity. Eight amino acid residues were identified as the key sites via molecular docking. Using a constructed screening library, we obtained the mutant DoxA(P88Y) with a more rational protein conformation, and a 56% increase in bioconversion efficiency was achieved by the mutant compared to the wild-type DoxA. Molecular dynamics simulation was applied to understand the relationship between the enzyme’s structural property and its substrate-binding efficiency. It was demonstrated that the mutant DoxA(P88Y) formed a new hydrophobic interaction with the substrate daunorubicin, which might have enhanced the binding stability and thus improved the catalytic activity. Our work lays a foundation for further exploration of DoxA and facilitates the industrial process of bio-production of doxorubicin.

## 1. Introduction

Doxorubicin, as one of the cytotoxic anthracycline agents, is a broad-spectrum anticancer drug, which is frequently used in the treatment of acute leukemia, malignant lymphoma, breast cancer, and other diseases [1]. Due to its high potency and moderate side effects, it is currently one of the most popular and effective anticancer medications [2]. Since doxorubicin cannot be manufactured in large quantities through microbial fermentation, its industrial production depends on the semi-chemical method using daunorubicin as its raw material [3,4]. However, product separation is more challenging due to the numerous by-products generated in the catalytic process, which raises production costs and causes environmental pollution. It is of great economic and application value to find a more efficient and clean method to produce doxorubicin.

**Figure 1:**
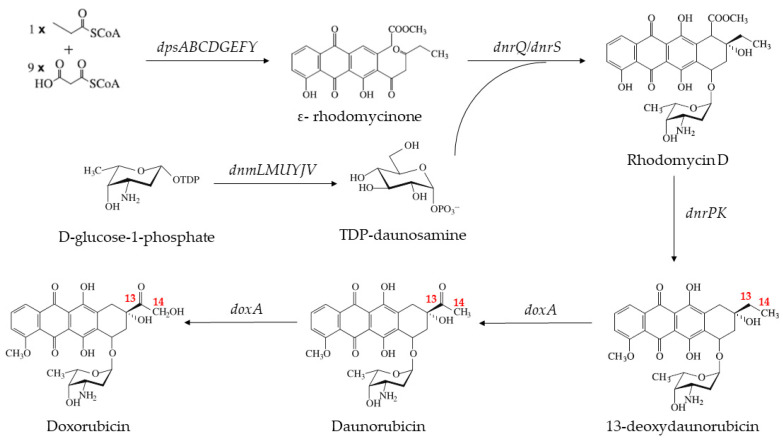
Biosynthesis pathway of doxorubicin.

Doxorubicin was first identified in the strain *Streptomyces peucetius* subsp. *caesius* ATCC 27952 in 1969, which was its sole producing strain [5]. Its synthetic pathway and the genes involved have been elucidated (Figure 1) [6]. Several previous studies focused on improving the rate of doxorubicin biosynthesis [7,8,9,10,11,12]. For instance, Malla et al. cloned the *dnrN*, *dnrI*, *afsR*, and *metK1-sp* genes from *Mycobacterium erythropolis* ATCC1 into the pIBR25 vector for expression, and eventually, the yield of doxorubicin was increased by 2.25–4.25-fold in some of the recombinant strains [11]. In the doxorubicin synthetic pathway, the cytochrome daunorubicin C-14 hydroxylase (DoxA), a member of the cytochrome P450 family, played an important role, which catalyzed the formation of daunorubicin from 13-deoxydaunorubicin and further oxidation to produce doxorubicin [13,14]. The final oxidation reaction was thought to be the rate-limiting step in the production of doxorubicin since the hydroxylation activity of DoxA at the C-14 position of daunorubicin was 170-fold lower than that at the C-13 position [15]. Therefore, the modification of DoxA to enhance the hydroxylation activity at the C-14 position of daunorubicin was critical to the efficient biosynthesis of doxorubicin.

For the molecular modification of proteins, protein engineering is a powerful tool to improve the specificity, regioselectivity, stereoselectivity, thermal stability, and solvent resistance of enzymes [16,17,18]. In it, various molecular modification strategies have been proposed, including directed evolution of enzymes, rational design, semi-rational design of proteins, and so on [19,20,21]. Therefore, a protein modification strategy could provide an interesting approach to the modification of DoxA [22].

The rational design of proteins can help to target the key amino acid residue sites affecting the binding efficiency of enzymes to substrates based on amino acid sequence analysis and three-dimensional structural information. Coupled with computer-aided screening, the efficiency of mutant protein screening could be greatly increased [21,23]. The rational design strategy has become an effective strategy for the efficient construction of screening libraries to obtain engineered enzymes with excellent performance [24,25]. For P450 enzymes, their rational design can improve the enzymatic activity and biocatalysis efficiency [26,27,28,29,30]. These studies have demonstrated the merits of the rational design strategy in P450 enzyme modification.

For rational design, molecular dynamics simulations and molecular docking are the commonly used techniques. Molecular dynamics simulations are used to understand the protein structure and function, which involves simulating the dynamics of the system and capturing its evolution over time in order to provide atomic-level parameters for the motion of ligand–receptor complexes [31,32]. In addition, it enables quantitative estimation of parameters that are not amenable to measurement in laboratory experiments, such as the torsional angle values to characterize flexibility, the solvent-accessible surface area to predict stability, and entropy variations for various structures [33,34]. Molecular dynamics simulations are currently applied at various stages of drug design and development, including assessing the potential biological activity of compounds in the virtual screening of ligands and evaluating the physicochemical and ADMET (absorption, distribution, metabolism, excretion, and toxicity) properties of compounds [35,36]. Molecular docking is also a general technique in the rational design process of proteins to predict the optimal binding of ligands in the corresponding receptors, depending on the free energy minimization of the ligand–receptor complexes [37]. However, most molecular docking procedures have limited flexibility to optimize the target, whereas molecular dynamics simulations can explore the receptor conformational space and generate a holistic image of the receptor conformation for further chemical database screening [38]. The results obtained through molecular docking can serve as a basis for molecular dynamics simulations [39].

In this study, the cytochrome P450 enzyme DoxA from *S. peucetius* subsp. *caesius* ATCC 27952 was heterologously overexpressed in *Escherichia coli*, and to improve its hydroxylation activity at the C-14 position of daunorubicin, a rational design strategy was applied to construct a powerful mutation library based on the crystal structure model of the DoxA protein and the molecular docking results. The optimum mutant DoxA(P88Y) was obtained with the highest efficiency for the conversion of daunorubicin to doxorubicin. Finally, molecular dynamics simulations were utilized to understand the relationship between the enzyme’s structural property and its substrate-binding efficiency. The work provided a theoretical basis for further modification of DoxA and improvement of its catalytic activity.

## 2. Results and Discussion

### 2.1. The Heterogenous Expression of DoxA and Analysis of Its Catalytic Activity

P450 enzymes are generally large in molecular weight and complex in structure, which leads to the easy formation of inclusion bodies when heterologously expressed in *E. coli* [40,41]. Therefore, the selection of an appropriate microbial host and optimization of expression conditions were crucial to achieving significant soluble expression of DoxA. In this study, the *DoxA* gene was PCR-amplified from the genomic DNA of *S. peucetius* subsp. *caesius* ATCC 27952 and assembled into the plasmid pET28a at the *Hin*dIII site (Supplementary Material, Figure S1). GroES and GroEL are commonly selected molecular chaperones to promote the expression of P450 enzymes [42,43]. The *E. coli* strain pGro7/BL21(DE3) harboring the molecular chaperones GroES and GroEL was selected as the host to introduce the recombinant plasmid pET28a-DoxA, which was induced at low temperature (16 °C) to promote the soluble expression of DoxA. Moreover, all P450 enzymes had a heme cofactor (iron protoporphyrin IX), which was necessary for oxygen binding to form the active catalytic center [44]. However, most microbial hosts had low heme concentrations. It has been reported that 5-aminolevulinic acid (ALA) as a precursor in the synthesis of heme could enhance the activity of P450 enzymes [45,46]. Therefore, 0.5 mmol/L ALA was added to the medium after isopropyl-β-D-thiogalactoside (IPTG) induction to enhance the activity of the expressed DoxA in our work.

According to the results of SDS-PAGE, an obvious peptide was found between 45 and 60 kDa in the supernatant sample after cell fragmentation, which was consistent with the molecule size of the DoxA protein (Supplementary Material, Figure S2). It was indicated that the *E. coli* pGro7/BL21(DE3) strain harboring the chaperone GroES/GroEL could effectively promote the correct folding of the protein and enhance the soluble expression of DoxA. After purification and concentration, the concentration of DoxA protein was up to 1673.8 mg/L as determined by the Bradford method, which was subsequently used to analyze the enzyme activity in vitro.

Wu et al. [47] successfully expressed DoxA by introducing the gene from *Streptomyces peucetius* ATCC 29050 and *Streptomyces coeruleorcbidus* SIPI 1482 into *Streptomyces lividans*, and daunorubicin was added to the fermentation medium during the cultivation process to explore the catalytic ability of DoxA. Although the expressed DoxA could achieve the conversion of daunorubicin to doxorubicin, the efficiency was very low (DoxA content was only 12% of the total protein content), and a lot of by-products were produced [47]. In our work, the catalytic activity of purified DoxA was also analyzed using daunorubicin as the substrate. According to the HPLC results, the peak at 12.956 min was considered to be daunorubicin, while the peak at 6.45 min was found to be doxorubicin by comparison with the results of the standards (Supplementary Material, Figure S3), which was also consistent with the retention time shown in the report [48]. It was indicated that the purified DoxA protein obtained in our work could catalyze the conversion of daunorubicin to doxorubicin, and the enzyme activity of heterologously expressed DoxA on the C-14 position of daunorubicin was calculated to be 0.286 U/mL. However, due to the poor specificity of DoxA, it also showed hydroxylation activity at other carbon positions and led to the formation of various by-products (Supplementary Material, Figure S3). The rational design strategy was thus considered for the modification of DoxA to improve its catalytic activity and specificity for the C-14 position of daunorubicin.

### 2.2. Protein Sequence Analysis and Three-Dimensional Model Construction of DoxA Based on I-TASSER

The Iterative Threading ASSEmbly Refinement (I-TASSER) server is evaluated as the best platform for protein structure and function prediction [49]. Odia and Adebiyi predicted the structure of CYP12F4 in the P450 protease family using computer techniques such as I-TASSER and 3D-BLAST, but the study of the structure of DoxA using these techniques has not been reported [50]. Due to the absence of experimental structural models, this study predicted the secondary structure and three-dimensional model of DoxA using I-TASSER based on the Local Meta-Threading Server (LOMETS). The top ten model templates with the highest predicted Z-score were selected according to the results of the iterative simulation by LOMETS (Supplementary Material, Figure S4). Generally, the amino acid sequence identity of the P450 enzyme family is less than 20%, and only three amino acids are completely conserved [51]. Therefore, there were no conserved sequences greater than 30% in the threading-aligned region (Iden1) and the entire template chain (Iden2) between the DoxA protein and the template protein. Additionally, according to the sequence alignment results in Figure S4, R31, T61, D99, H103, L133, P147, I163, L166-P170, V218, V293-E295, R298, R309, V315, N336, D338, R352, G362, G364, H366, C368, F388, and P389 were completely conserved residues that might be important catalytic sites for protein–substrate binding.

**Figure 2:**
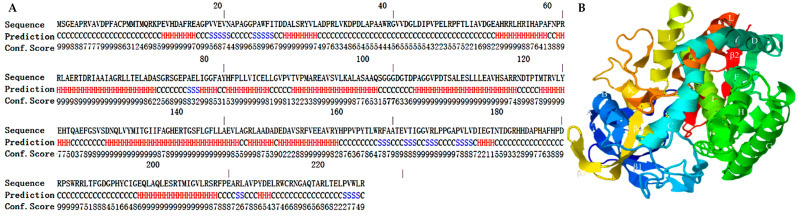
The secondary structure and three-dimensional model of DoxA predicted by I-TASSER. (**A**): the secondary structure of DoxA (H was the α helix; S was the β strand; C was the random coil), (**B**): the three-dimensional model of DoxA.

The secondary structure and the three-dimensional model of the DoxA protein predicted by the I-TASSER server are shown in Figure 2. It was indicated that the DoxA sequence contained 12 α-helices (A-K) and 4 β-folds (β1-β4). According to the reports, the P450 family usually consists of two major structural blocks: a β-sheet rich region on one side and an α-helix rich region on the other, while the α-long helices I located across the center was regarded as the highly conserved region [52,53], and the three-dimensional model of DoxA predicted in our work was consistent with these characteristics. Meanwhile, the C score (confidence score) and TM score (template modeling score) are important parameters reflecting the quality of the model [54]. The C score indicates the confidence level of the template and model, which should be in the range of [−5, 2] [54], and a higher C-score value demonstrates a higher confidence level of the model. The C score of the DoxA model predicted in our work was 0.27, which meant that the model had a high level of confidence. The TM score represents the structural similarity between the predicted model and the native structure usually with a value in the range of [0, 1] [55]. A higher TM score indicates a better structural match. The TM score of the DoxA model was 0.75 ± 0.10, which proved that the model had high quality and credibility.

A heme cofactor (iron protoporphyrin IX) was contained in the interior of all the P450 enzymes, which acted as the active center in the catalytic process [56]. A complete three-dimensional model of DoxA should contain the heme ligand. However, there were few reports on the binding sites of heme ligands in P450 proteins. 3DLigandSite is a web server for the prediction of ligand-binding sites. It can provide structural models for proteins based on structure prediction, and ligands can be superimposed onto the model to predict the binding sites [57]. Therefore, the 3DLigandSite server was applied in our work to predict the binding sites of the heme ligand in DoxA. The iron atom at the center of heme was reported to process six coordination sites, one of which was linked to the nearby cysteine or histidine residue [58]. According to the predictions of the 3DLigandSite server, 367Cys residue of DoxA was most likely the binding site of the heme ligand. It was essential to ensure that the distance was within the normal electrostatic interaction force range (about 1–2 Å) between the iron atom of heme and the 367Cys residue, which could avoid spatial conflicts with the other amino acids. The complete DoxA-heme three-dimensional model was thus determined by superimposing the heme ligand onto the model (Supplementary Material, Figure S5).

### 2.3. Identification of the Mutant Sites and Establishment of Screening Libraries

**Figure 3:**
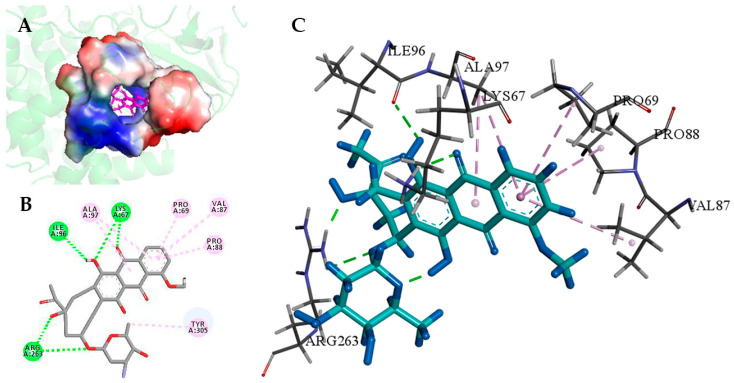
Docking pose of daunorubicin in DoxA-heme model. (**A**): daunorubicin binding pocket conformation, (**B**,**C**): drawing of the mechanism of the daunorubicin-binding region and the amino acid residues interacting with the daunorubicin molecule.

Based on the complete DoxA-heme three-dimensional model, the molecular docking of the substrate daunorubicin into the model was subsequently performed using the docking program GOLD. According to the docking results in Figure 3, the binding sites of daunorubicin to DoxA-heme were located at the center of the protein structure and were mainly dependent on hydrogen bonds and π-alkyl hydrophobic interactions. The amino acid residues 67Lys, 96Ile, and 263Arg could form hydrogen bonds with daunorubicin, while the residues 69Pro, 87Val, 88Pro, 97Ala, and 305Tyr could form hydrophobic interactions with daunorubicin. In addition, the four residues 69Pro, 87Val, 88Pro, and 97Ala binding to the first benzene ring of daunorubicin were considered the key sites in the binding pocket region (Figure 3).

Previously, Ba et al. [29] applied the semi-rational design to modify the P450_sca-2_ enzyme from *Streptomyces carbophilus*, and five amino acid residues R77, T85, V194, T119, and N363 were identified as the essential substrate-binding sites using the Discovery Studio (LibDock). The residues T85 were located in the binding pocket, and mutation of T85 to methionine and leucine was found to increase the hydrophobicity of the substrate-binding pocket, which led to a significant enhancement in the expression and catalytic efficiency of P450_sca-2_ [29]. Similarly to T85, the amino acid residues docked to daunorubicin above were located in the binding pocket region of DoxA. They were thus focused on conducting the subsequent mutation library construction and computer-aid screening.

The most thermodynamically stable protein has the lowest conformational free energy. The protein conformation of the DoxA-heme model was firstly pre-optimized by the Rosetta Cartesian_ddG operator and yielded 20 protein conformations with different free energy scores (Supplementary Material, Table S1). The doxA_I-TASSER_0019 with the lowest free energy was thus selected as the initial conformation. Based on the molecular docking results, eight amino acid residues in the DoxA binding pocket were mutated to several different amino acids with different hydrophobicity to construct the mutation library. In addition, available mutants were screened by calculating the free energy difference (ΔΔG) between the mutant and wild-type DoxA. As shown in Table 1, the mutants DoxA(P69Y), DoxA(V87A), DoxA(V87L), DoxA(P88Y), and DoxA(P88T) were the optimum mutants, showing lower free energy compared with the wild-type DoxA and exhibiting a relatively large change in ΔΔG (−6.10, −8.13, −9.04, −12.24, and −8.06). It implied that these five mutants had a more rational protein conformation, which might improve the substrate-binding stability of DoxA and thus the catalytic efficiency.

Therefore, five new plasmids containing these five DoxA mutants were constructed upon the sequence verification (Supplementary Material, Figure S6), and they were subsequently transformed into *E. coli* pGro7/BL21 (DE3) for enzyme expression and purification. SDS-PAGE electrophoresis results showed that all the mutant enzymes were well expressed (Supplementary Material, Figure S7), and the purified-concentrated protein concentrations reached 6.38 g/L, 1.47 g/L, 5.52 g/L, 2.94 g/L, and 5.54 g/L, respectively, all higher than the concentration range (49–335 nmol/L) measured by Nakamura et al. for the P450 2A6 mutant [59]. This facilitated further determination of their enzymatic activities in the conversion of daunorubicin to doxorubicin.

**Figure 4:**
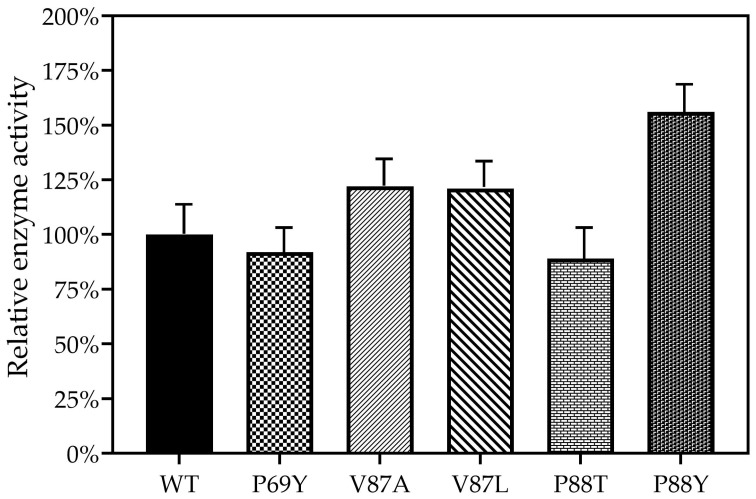
The relative enzyme activities of wild-type DoxA and the mutants.

As shown in Figure 4, the relative enzyme activity was defined as 100%. It was found that the enzyme activities of the mutants DoxA(P69Y) and DoxA(P88T) decreased, while the activities of DoxA(V87A), DoxA(V87L), and DoxA(P88Y) were enhanced, showing 22%, 21%, and 56% increases in the conversion efficiency of daunorubicin to doxorubicin compared with the wild-type DoxA, respectively. The residue 87V was a strongly hydrophobic amino acid. When it was mutated to the less hydrophobic amino acids, such as alanine and isoleucine, the enzyme activity increased rather than decreased. It was speculated that the hydrophobicity of V87A and V87L did not weaken their hydrophobic interactions with the substrate. The residue 88P was a weak nonpolar hydrophobic amino acid. It was interesting that when 88P was mutated to the hydrophobic amino acids, such as tyrosine and serine, the enzymatic activity showed an increasing and decreasing trend, respectively. It might indicate that there was no apparent correlation between the hydrophobicity or hydrophilicity of amino acids, the binding sites of substrates, and the changes in enzyme activity.

### 2.4. Understanding the Relationship between the Enzyme Structural Property and Its Substrate-Binding Efficiency

**Figure 5:**
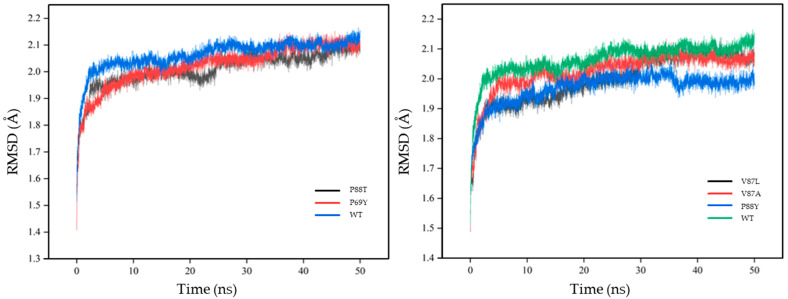
The root-mean-squared deviation (RMSD) values of different protein–ligand complexes.

**Figure 6:**
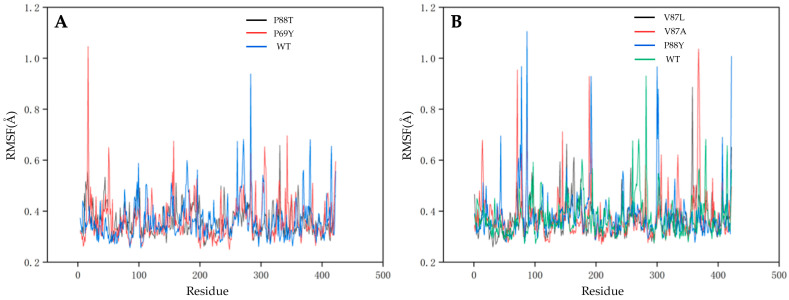
The root-mean-squared fluctuation (RMSF) values of each residue of proteins. (**A**): P88T and P69Y; (**B**): V87L, V87A and P88Y.

In a previous study, Bathelt et al. analyzed the electronic structure of compound I in the P450 heterodimer using QM/MM simulations that better described the reaction process and helped to further analyze the metabolism of the relevant drug [60]. In this work, GROMACS, a software suitable for simulating macromolecular dynamics, was chosen to evaluate the stability and dynamic properties of the wild-type DoxA and five mutants (DoxA(P69Y), DoxA(P88T), DoxA(V87A), DoxA(V87L), and DoxA(P88Y)) binding with the substrate daunorubicin at the temperature of 300 K. The root-mean-square deviation (RMSD) values were examined during 50 ns of simulation, and the results are shown in Figure 5. At the temperature of 300 K, the RMSD values of almost all proteins reached the equilibrium after 10 ns, and the values were maintained at about 2 Å without significant fluctuations, indicating the fast formation and high stability of protein–substrate complexes. Moreover, compared with the wild-type DoxA, all the mutants had lower RMSD values, which meant that their trajectories were less skewed and the mutation of DoxA promoted the overall structural stability of the protein–substrate complex. The dynamic root-mean-square fluctuation (RMSF) value is also an important indicator of residue flexibility during molecular dynamics simulations. The RMSF values of wild-type DoxA were also compared with those of five mutants (Figure 6). It was shown that the RMSF values of the wild-type DoxA were similar to those of the mutant DoxA(P69Y) and DoxA(P88T), but the values of the β1 fold region (residues 37–53) of both mutants were significantly higher than those of the wild-type DoxA (Figure 6A), which meant that the mutation of P69Y and P88T attenuated the overall rigidity of the β1 region. It might be due to the increased resistance of the spatial site in this region after the mutation, which caused a displacement of the β1 fold structure and led to the weakness of the regional stability and flexibility upon binding to the substrate daunorubicin. Furthermore, the RMSF values of the mutants DoxA(V87A), DoxA(V87L), and DoxA(P88Y) also showed a similar variation trend to those of the wild-type DoxA (Figure 6B). Meanwhile, the RMSF values of these three mutants in the αI helix (residues 262–276) near the α-helix structure-rich region of heme were lower than those of the wild-type DoxA, which demonstrated that the rigidity of the αI helix region was increased. Since the αI helix was the longest α-helix structure running through the center of DoxA, the improvement of its rigidity could promote the stability of the protein and thus enhance the catalytic activity of the enzyme. Based on these results, it was hypothesized that the point mutations in the protein did not affect the stability of the mutated region, but rather the stability of the secondary structures such as α-helix and β-fold, which in turn had a significant impact on the catalytic activity of the enzyme.

Besides the stability and dynamic properties of proteins, the binding free energy is also one of the most important qualities to reflect the protein–ligand binding affinities [61]. Molecular mechanics Poisson-Boltzmann surface area (MM-PBSA) is an arguably very popular method for binding free energy prediction [62]. It was thus combined with molecular dynamics simulations to calculate the binding free energies of the mutants with the substrate. The binding free energy (ΔG_binding_) consisted of the gas-phase free energy (G_gas_) and solvent-phase free energy (G_solv_), which were contributed from van der Waals interaction energy (G_van_), electrostatic interaction energy (G_ele_), nonpolar solvation energy (G_np_) and polar solvation energy (G_pol_). As shown in Table 2, the electrostatic interactions and the polar solvation interactions were the major factors impacting the binding free energy of all the proteins. Both wild-type DoxA and the mutants had favorable G_ele_ values in the range of −227.14 to −304.01 kcal/mol and unfavorable G_pol_ values in the range of 224.04 to 289.08 kcal/mol. It was indicated that the association was unbeneficial for the unfavorable desolvation of polar groups. Due to the influence of the factors above, the final binding free energies of DoxA and the mutants showed different changes. Compared with the wild-type DoxA, the mutants DoxA(V87A), DoxA(V87L), and DoxA(P88Y) had lower ΔG_binding_ values, while DoxA(P69Y) and DoxA(P88T) had higher ΔG_binding_ values, which was consistent with the changes in enzyme activity.

To further investigate the effect of point mutations on amino acid residues, the decomposition of binding free energies into contributions from individual residues was analyzed (Table 3). It was shown that the changes in the total energy of these three amino acid sites were almost consistent with the changes in enzyme activity of wild-type DoxA and the mutants, and the total energy of the three amino acid residues in mutant DoxA(P88Y) was the lowest. It proved that these three amino acid residues played a favorable role in the formation of the protein–substrate complex and the mutation of these residues impacted the binding free energy, thereby affecting the enzyme activity.

**Figure 7:**
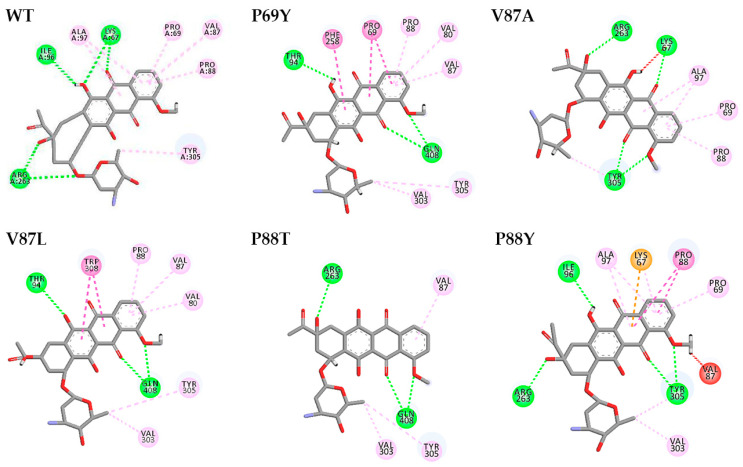
Analysis of the binding modes of daunorubicin to wild-type DoxA/mutants via GOLD docking.

The GOLD program can perform flexible protein–ligand docking to predict the interaction of the substrate with protein and test the quality of protein–ligand complexes [63]. This work was the first to analyze the binding modes of ligands in wild-type DoxA and the mutants using the GOLD program. According to the molecular docking results above, hydrophobic interactions were the main force during the binding of daunorubicin to DoxA. As shown in Figure 7, the GOLD docking results indicate that the first benzene ring remained the primary binding site for the substrate to the enzyme, which meant that the point mutation did not alter the major interaction force between the mutants and the substrate. The mutant DoxA(P88Y) mutating the residue to tyrosine with a phenyl ring resulted in the formation of π–π interactions with the substrate, which suggested that mutating the branch structure of amino acids could cause the formation of new hydrophobic interactions at the binding site and enhance the interaction of the substrate with the protein. It was thus concluded that the point mutation did not affect the hydrophobic interaction of the enzyme with the substrate, and this interaction was further reinforced in the mutant DoxA(P88Y), thereby leading to the increase in enzyme activity.

## 3. Materials and Methods

### 3.1. Strains and Cultivation

*E. coli* DH5α (purchased from Tsingke Biotechnology Co., Ltd., Beijing, China) was used for plasmid construction and maintenance, while *E. coli* pGro7/BL21 (DE3) (purchased from Novoprotein Scientific Inc., Suzhou, China) was used as the host to express DoxA. *S. peucetius* subsp. *caesius* ATCC 27952 was used to prepare the genome as the template for cloning the *DoxA* gene.

The ISP medium containing tryptone 5 g/L, yeast extract 3 g/L, and 15 g/L agar was prepared for *S. peucetius* subsp. *caesius* ATCC 27952 cultivation, which was incubated at 26 °C for 72 h to obtain single colonies. Then, the single colonies were transferred to shake flasks containing50 mL NDYE medium (maltose 22.5 g/L, yeast extract 5.04 g/L, sodium nitrate 4.28 g/L, KH_2_PO_4_ 0.23 g/L, HEPES 4.77 g/L, MgSO_4_·7H_2_O 0.12 g/L, NaOH 0.4 g/L, trace element solution 2 mL/L (ZnCl_2_ 40 mg, FeCl_3_·6H_2_O 200 mg, CuCl_2_·2H_2_O 10 mg, MnCl_2_·4H_2_O 10 mg, Na_2_B_4_O_7_·10H_2_O, 4MoO_3_.3H_2_MoO_4_·4H_2_O.6H_3_N 10 mg)), and cultivated at 26 °C, 180 rpm for 72 h to collect cells for genome preparation. LB broth (5 g/L yeast extract, 10 g/L NaCl, and 10 g/L tryptone) and an agar plate with 50 μg/mL kanamycin were used to cultivate the engineered *E. coli*. After being transformed into the plasmid, *E. coli* pGro7/BL21 (DE3) was cultivated on the LB plate overnight at 37 °C to obtain single colonies, which were then transferred into tubes containing 4 mL LB broth and incubated at 37 °C, 220 rpm for 12~16 h to finish the seed cultivation stage. Subsequently, the seed solution was inoculated into 500 mL LB broth at a ratio of 1:100, and 2 g/L L-arabinose was added to the broth as the extra carbon source. After being cultivated at 37 °C, 220 rpm for 3 h, the OD600 reached about 0.6~0.8. Then, 0.2 mmol/L IPTG and 0.5 mmol/L 5-aminolevulinic acid was added to the broth, which was then cultivated at 16 °C, 180 rpm for 48 to obtain the cells with DoxA expressed.

### 3.2. Plasmid Construction

Plasmid pET28a (purchased from Sangon Biotech (Shanghai, China) Co., Ltd.) was used as the backbone vector. DoxA gene was PCR-amplified from the genome of *S. peucetius* subsp. *caesius* ATCC 27952 with the primers (Supplementary Material Table S2). Due to a high GC content (over 70%) of the genomic DNA, the hydrogen bonds between the double chains were strong, and the PCR amplification required the addition of 5% dimethyl sulfoxide to promote the denaturation of the template and enhance the specificity of binding to the primers. The final fragment length of the *DoxA* gene was about 1.3 kb and was inserted into the pET28a vector at the *Hin*dIII site to generate the plasmid pET28a-DoxA. The five mutants of DoxA were obtained via PCR based on the Fast Mutagenesis System spot mutagenesis kit (purchased from TransGen Biotechnology Co., Ltd., Beijing, China). The restriction enzymes, the plasmid miniprep, PCR clean-up, and gel DNA recovery kits used in our work were purchased from TransGen Biotechnology Co., Ltd.

### 3.3. Enzyme Purification

At the end of the fermentation process, cells were harvested in a 50 mL tube by centrifugation (6600× *g*, 10 min, −4 °C) and then resuspended in 10 mL sterilized water and centrifuged (6600× *g*, 10 min, −4 °C) to remove supernatant. The obtained cells were finally resuspended in 5 mL lysis buffer (NaH_2_PO_4_ 50 mmol/L, NaCl 300 mmol/L, imidazole 10 mmol/L, glycerol 10%, pH 8.0) and disrupted by ultrasonication at 200 W for 10 min. The cell disruption suspension was centrifuged (8000× *g*, 4 min, −4 °C), and the supernatant was filtered through a 0.22 μm microporous membrane to obtain crude enzyme of DoxA. The crude enzyme was further purified using a HisTALON column (Takara, San Jose, CA, USA) [64]. The protein concentration of the purified enzyme was determined by the Bradford method [65].

### 3.4. Enzyme Activity Analysis

The activity of the purified DoxA was determined in vitro. The reaction system consisted of the purified DoxA enzyme (1 μmol/L), spinach ferredoxin (5 μmol/L), spinach ferredoxin reductase (1 μmol/L), glucose-6-phosphate (4 μmol/L), glucose-6-phosphate dehydrogenase (5 U), peroxidase (100 ng), magnesium chloride (10 mmol/L), daunorubicin (100 μmol/L), and NADH (250 mmol/L, dissolved in 50 mmol/L sodium phosphate buffer). The volume of the reaction system was made up to 100 μL by adding sterilized water, and the reaction was conducted at 30 °C for 2 h. Afterward, the reaction was terminated by adding 100 mmol/L Tris-buffer. The reaction solution was extracted with the methane/chloroform solution (1:9, *v*/*v*), dried with nitrogen gas blow, and then re-dissolved in 100 μL methanol for the analysis. The analysis was performed using HPLC with a UV detector at the wavelength of 254 nm. A solution containing 56% methanol, 41% water, 2.5% acetic acid, and 0.5% triethylamine was used as the mobile phase with a flow rate of 1 mL/min. Hypersil ODS2 5 μm was the column with a detection temperature of 35 °C.

### 3.5. Molecular Docking Analysis

The secondary structure and initial three-dimensional model of DoxA were predicted via the I-TASSER server based on the amino acid sequence. The 3DLigandSite server was applied to predict the binding sites of the heme ligands in DoxA to obtain the complete DoxA-heme three-dimensional model. The molecular docking of the substrate daunorubicin into the DoxA-heme model was performed using the docking program GOLD. The PDB file of DoxA-heme was operated in GOLD to remove water and other small molecules and add hydrogen atoms. Twenty flexible residues within 6 Å of the hydrophobic pocket region were selected as active sites. The critical amino acid residues were identified according to the GOLD score and hydrogen bond interaction.

### 3.6. The Calculation of Conformational Free Energy

Based on the key amino acid residues identified by the molecular docking, the screening mutation library of DoxA was established and the Rosetta Cartesian_ddG program was used to calculate the conformational free energy changes (ΔΔG) caused by the mutation modes of different amino acid residues so as to screen the relatively rational protein conformations.

### 3.7. Molecular Dynamics Simulation

The 100 ns molecular simulations of wild-type DocA/mutants were performed using GROMACS 5.1.2 with the GROMOS96 43A1 force field. The topology of the protein and ligand was generated using pdb2gmx and the PRODRG server [66]. The ligand was combined in the complex topology files with all hydrogen atoms being added. The models were soaked in a cubic box for setting boundary conditions, and the protein was subjected to a solvate in the Simple Point Charge water model. Na^+^ ion was added to the complexes for neutralizing, and the system was minimized using 1500 steps of steepest descent. After equilibration in two phases (NVT (Constant Number Particles, Volume, and Temperature) and NPT (Constant Number Particles, Pressure, and Temperature), the system reached a suitable temperature and pressure, and the resulting trajectories were analyzed using the utilities of GROMACS. AmberTools16 was used for the calculation of trajectories and Pymol was used for graphical presentation.

### 3.8. MM-PBSA Binding Free Energy Calculation

MM-PBSA (molecular mechanics Poisson–Bolzmann surface area) is a method for post-processing the molecular dynamics trajectories to evaluate the binding free energy [62]. The binding free energy of five protein–ligand complexes was calculated via MM-PBSA protocols using the g_mmpbsa tool [67]. The following equations were used to calculate the binding free energies (Δ*G_binding_*) of the complexes.

$$\Delta G_{binding}=G_{binding}-G_{free-protein}-G_{free-ligand}$$

where *G_binding_* means the total free energy of the binding complex, and *G_free-protein_* and *G_free-ligand_* are the total free energies of the individual protein and ligand, respectively.

## 4. Conclusions

DoxA is the essential enzyme catalyzing the production of doxorubicin from daunorubicin, which is important for the large-scale enzymatic synthesis of doxorubicin. In this work, the DoxA from *S. peucetius* subsp. *caesius* ATCC 27952 was successfully overexpressed in the host *E. coli* pGro7/BL21 (DE3) with a good capacity in the conversion of daunorubicin in vitro after the optimization of the cultivation conditions. To further improve the catalytic activity, a rational design strategy was applied to modify DoxA. Firstly, a widely available method for efficient construction of the complete protein structure of cytochrome P450 enzymes was obtained using I-TASSER and the 3DLigandSite online server. The structure was found to be similar to that of the P450 enzyme family and consisted of an α-helix enriched region and a β-fold enriched region. Secondly, the molecular docking of the substrate daunorubicin into the DoxA-heme model was performed using the docking program GOLD, which provided a theoretical basis for further activity enhancement on the basis of DoxA mutant enzymes with improved catalytic activity. Eight amino acid residues (67Lys, 96Ile, 263Arg, 69Pro, 87Val, 88Pro, 97Ala, and 305Tyr) in the binding pocket of DoxA were identified as the key sites and mutated to several amino acids with different hydrophobicity to construct the screening library. Upon the calculation of the free energy, five mutants (DoxA(P69Y), DoxA(P88T), DoxA(V87A), DoxA(V87L), and DoxA(P88Y)) with lower free energy were considered to have more rational protein conformations. Afterward, the five mutants were overexpressed in *E. coli* pGro7/BL21 (DE3) to analyze their catalytic activities, and the mutant DoxA(P88Y) was the optimum, showing a 56% increase in the conversion efficiency of daunorubicin to doxorubicin. Finally, molecular dynamics simulations were utilized to understand the relationship between structural properties and substrate-binding efficiency. It was demonstrated that the mutants had better stability and lower binding free energy to the substrate compared with wild-type DoxA. Meanwhile, the point mutation did not affect the primary interaction forces upon binding to the substrate, and the mutant DoxA(P88Y) formed new hydrophobic interactions with the substrate, which enhanced the binding stability of the substrate to the enzyme, thus exhibiting higher catalytic activity. These findings provided a potential research foundation and theoretical rationale for further improving the catalytic activity of DoxA. In the future, the substrate specificity of the mutant DoxA(P88Y) still needs to be improved and further analyzed for other hydroxylation sites of daunorubicin in addition to C-14. It is expected that a combinatorial mutagenesis strategy will be used to further enhance the enzyme’s catalytic activity.

## Figures and Tables

**Table 1:** The calculation of the free energy difference (ΔΔG, kcal/mol) between the mutants and wild-type DoxA.

Mutants	ΔΔG	Mutants	ΔΔG
DoxA(I96L)	−5.12	DoxA(P88Y)	−12.24
DoxA(I96V)	0.92	DoxA(P88T)	−8.06
DoxA(I96F)	−1.94	DoxA(K67A)	4.11
DoxA(Y305V)	2.76	DoxA(K67R)	0.72
DoxA(Y305F)	0.68	DoxA(K67H)	4.70
DoxA(Y305W)	−1.01	DoxA(A97L)	3.52
DoxA(P69V)	0.02	DoxA(A97I)	2.36
DoxA(P69Y)	−6.10	DoxA(A97T)	1.39
DoxA(P69T)	−0.49	DoxA(L67I)	4.68
DoxA(V87A)	−8.13	DoxA(L67A)	4.58
DoxA(V87I)	−2.86	DoxA(L67S)	3.61
DoxA(V87L)	−9.04		

**Table 2:** Calculation of the binding free energy (kcal/mol) of wild-type DoxA and the mutants.

Binding Free Energy	DoxA	DoxA (P69Y)	DoxA (V87A)	DoxA (V87L)	DoxA (P88T)	DoxA (P88Y)
G_van_	−54.10	−58.04	−57.62	−58.48	−57.54	−52.90
G_ele_	−257.77	−277.60	−304.01	−294.50	−227.14	−277.32
G_pol_	248.42	272.77	289.08	285.83	224.04	262.99
G_np_	−7.58	−8.03	−7.65	−7.27	−7.88	−7.44
G_gas_	−311.87	−335.64	−361.63	−352.98	−284.68	−330.22
G_solv_	240.84	264.74	281.43	278.56	216.16	255.55
ΔG_binding_	−71.03	−70.90	−80.20	−74.42	−68.52	−74.67

**Table 3:** Decomposition of binding free energy (kcal/mol) into contributions from individual residues.

Residues	DoxA	DoxA (P69Y)	DoxA (V87A)	DoxA (V87L)	DoxA (P88T)	DoxA (P88Y)
Pro 69	−0.83	−0.51	−0.48	−0.53	−0.37	−0.82
Val 87	−0.29	−0.30	−0.29	−0.80	−1.21	−0.95
Pro 88	−0.02	−0.23	−0.89	−0.05	−0.02	−0.10
The total	−1.14	−1.04	−1.66	−1.38	−1.60	−1.87

## Data Availability

The data that support the findings of this study are available from the corresponding author upon reasonable request.

## Supplementary Materials

The supporting information can be downloaded at: https://www.mdpi.com/article/10.3390/ijms24098337/s1.
