# Supplementary material for: Rational Design of Daunorubicin C-14 Hydroxylase Based on the Understanding of Its Substrate-Binding Mechanism
Source: Int J Mol Sci. 2023 May 6;24(9):8337. doi: 10.3390/ijms24098337 (PMC10179135; doi:10.3390/ijms24098337)
Supplement: Supplementary file 1 [file ijms-24-08337-s001.zip › ijms-2355819-supplementary.pdf]

## Supplementary data

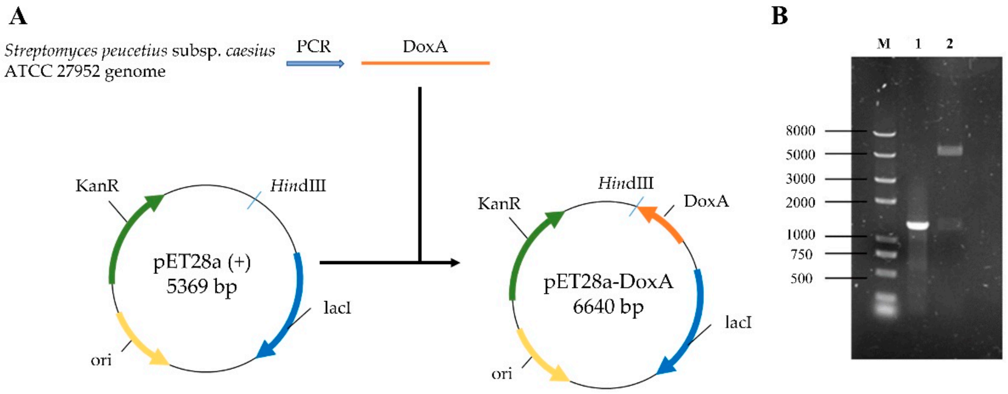


**Figure S1.** Schematic diagram of the recombinant plasmid pET28a-DoxA construction. (**A**): The construction of recombinant plasmid pET28a-DoxA; (**B**): DNA Electrophoresis profile of DoxA. Lane M: DNA marker, lane 1: *doxA* gene, lane 2: enzyme digestion of pET28a-DoxA.

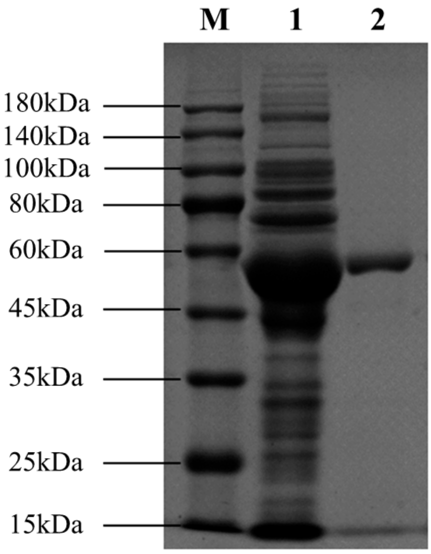


**Figure S2.** SDS-PAGE protein profile of DoxA. Lane M: protein marker, lane 1: the supernatant sample after cell fragmentation, lane 2: the purified DoxA.

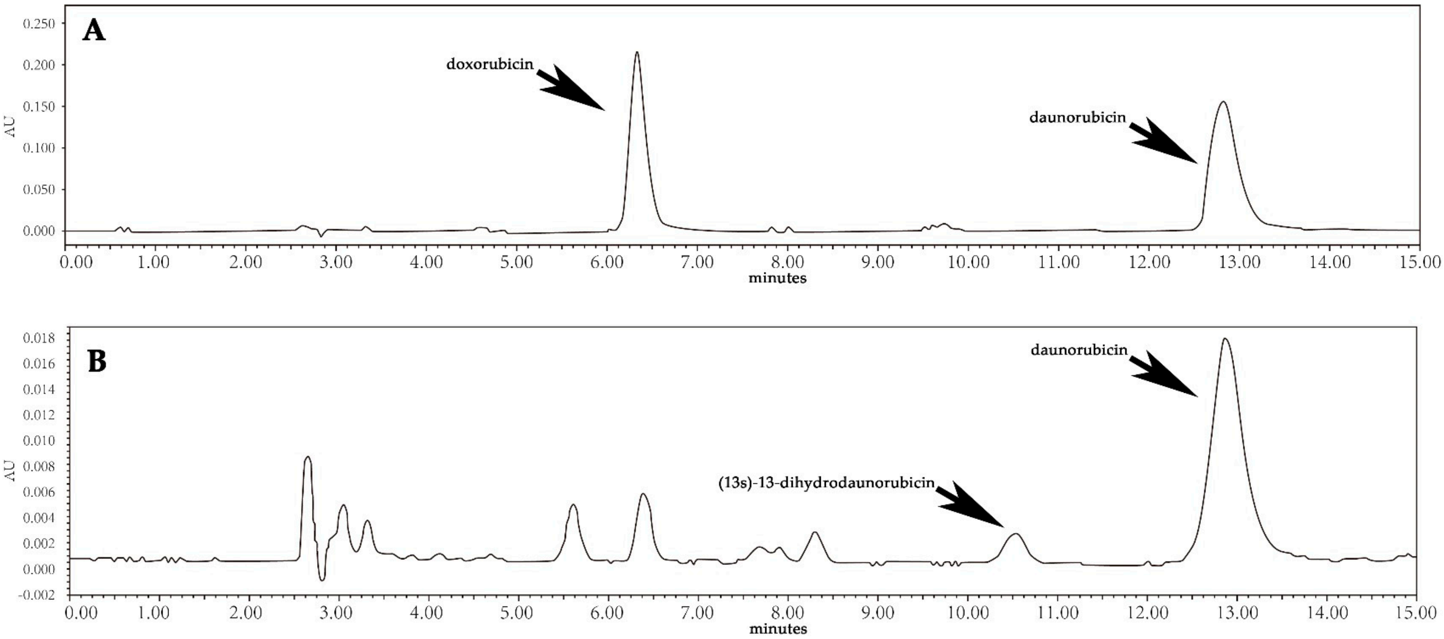


**Figure S3.** HPLC analysis of the catalytic products of daunorubicin by DoxA. (**A**): HPLC results of the doxorubicin/daunorubicin standards; (**B**): HPLC result of the sample.

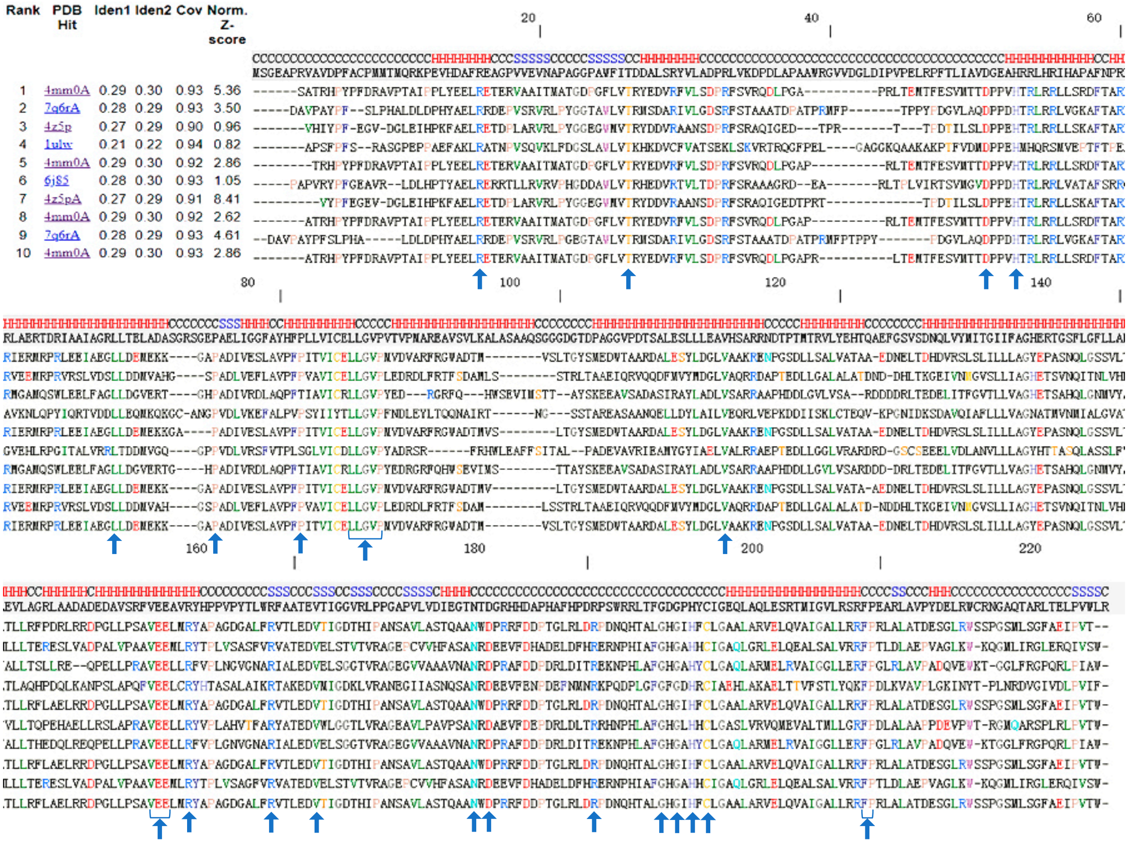


**Figure S4.** Multiple sequence alignment of DoxA with other homologous enzymes by LOMETS. (**Notes**: The blue arrows were the conserved residues that might be important catalytic sites for protein-substrate binding.)

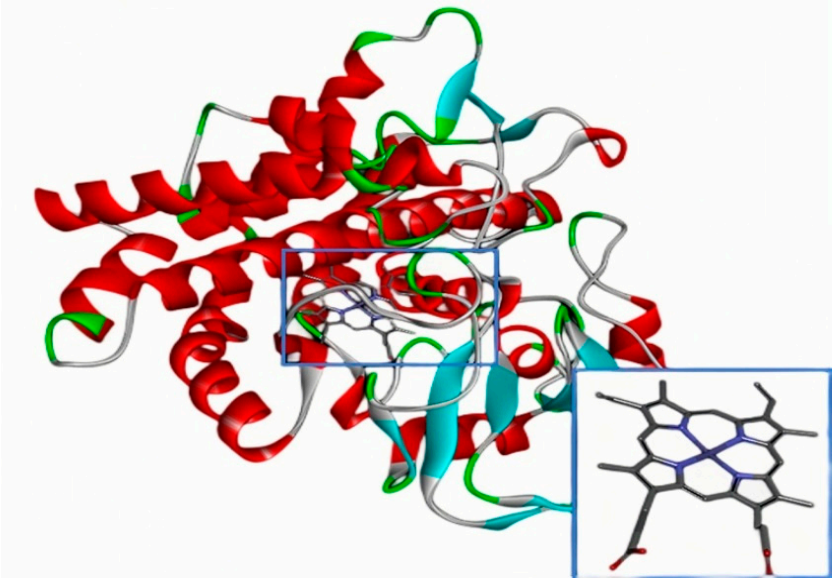

**Figure S5.** The complete DoxA-heme three-dimensional model.

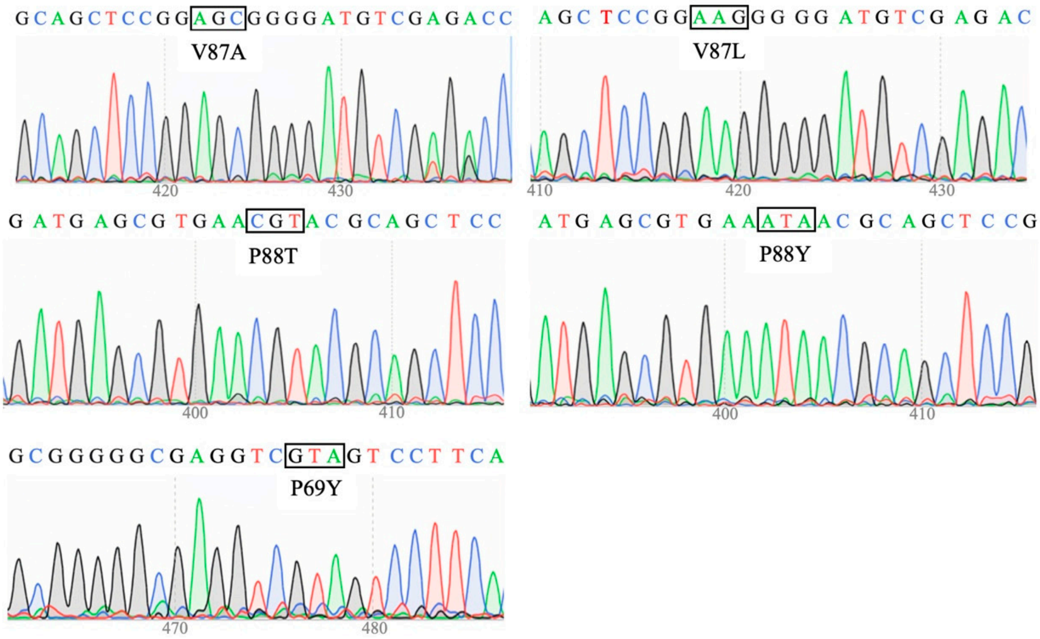


**Figure S6.** Sequence verification of the five plasmids containing DoxA mutants. (**Notes**: The black boxes were the mutant sites in the sequence of DoxA.)

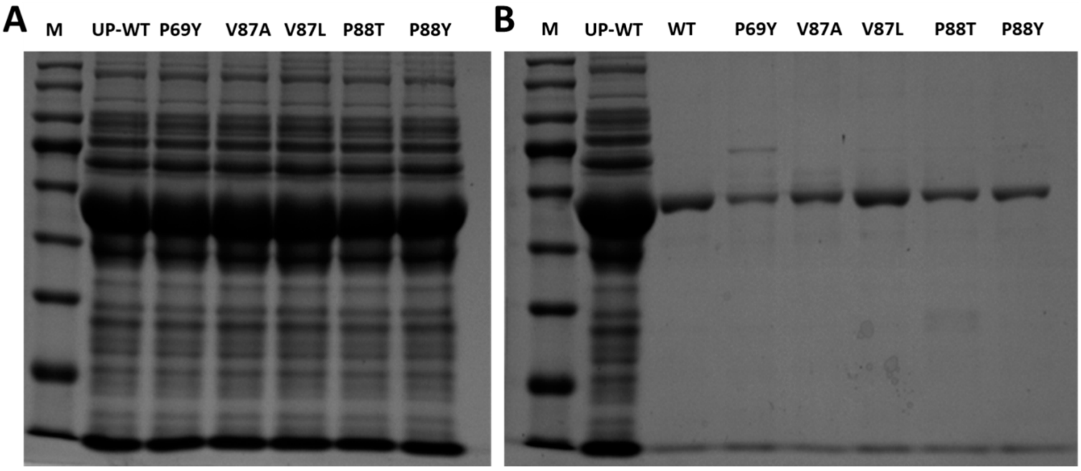


**Figure S7.** SDS-PAGE protein profile analysis of the mutants of DoxA. (**A**): the supernatant samples after cell fragmentation; (**B**): the purified proteins (Lane M: protein marker, lane UP-WT: unpurified wild-type DoxA, lane WT: purified wild-type DoxA).

**Table S1.** Free energy of DoxA-model with different protein conformations

| Name | Score |
|---|---|
| doxA_I-TASSER_0001 | -695.393 |
| doxA_I-TASSER_0002 | -672.837 |
| doxA_I-TASSER_0003 | -664.821 |
| doxA_I-TASSER_0004 | -708.753 |
| doxA_I-TASSER_0005 | -663.290 |
| doxA_I-TASSER_0006 | -706.663 |
| doxA_I-TASSER_0007 | -671.219 |
| doxA_I-TASSER_0008 | -700.996 |
| doxA_I-TASSER_0009 | -672.137 |
| doxA_I-TASSER_0010 | -669.950 |
| doxA_I-TASSER_0011 | -637.247 |
| doxA_I-TASSER_0012 | -702.992 |

| | |
|---|---|
| doxA_I-TASSER_0013 | -672.706 |
| doxA_I-TASSER_0014 | -644.515 |
| doxA_I-TASSER_0015 | -705.554 |
| doxA_I-TASSER_0016 | -701.584 |
| doxA_I-TASSER_0017 | -676.329 |
| doxA_I-TASSER_0018 | -658.026 |
| doxA_I-TASSER_0019 | -716.475 |
| doxA_I-TASSER_0020 | -672.129 |

**Table S2.** Primers used in this work

| **Primers** | **Sequence 5′-3′** |
|---|---|
| DoxA-F | **TCGAGTGCGGCCGCAAGCTTGCGCAGCCAGACGGGC** |
| DoxA-R | **CGAGCTCCGTCGACAAGCTATGAGCGGCGAGGCGC** |
| PET-F | **GATCTCGATCCCGCGAA** |
| PET-R | **GCAGCCAACTCAGCTTC** |
| P69Y-F | **GGCGGGGGCGAGGTCGTAGTCCTTCACG** |
| P69Y-R2 | **TACGACCTCGCCCCCGCCGCCTGGCGGG** |
| V87A-F | **CGGACGCAGCTCCGGAGCGGGGATGTCG** |
| V87A-R | **GCTCCGGAGCTGCGTCCGTTCACGCTCA** |
| V87L-F | **CGGACGCAGCTCCGGAAGGGGGATGTCG** |
| V87L-R | **CTTCCGGAGCTGCGTCCGTTCACGCTCA** |
| P88Y-F | **GGCGATGAGCGTGAAATAACGCAGCTCC** |
| P88Y-R | **TATTTCACGCTCATCGCCGTGGACGGCG** |
| P88T-F | **GGCGATGAGCGTGAACGTACGCAGCTCC** |
| P88T-R | **ACGTTCACGCTCATCGCCGTGGACGGCG** |
